# Air Leakage Characteristics of Gas Boreholes in Deep Coal Seams and Application of Short-Hole Grouting and Plugging

**DOI:** 10.1021/acsomega.4c00331

**Published:** 2024-05-07

**Authors:** Fan Wu, Shibin Wang, Cheng Fan, Yueping Qin, Xiangyun Su

**Affiliations:** School of Emergency Management and Safety Engineering, China University of Mining and Technology-Beijing, Beijing 100083, P. R. China

## Abstract

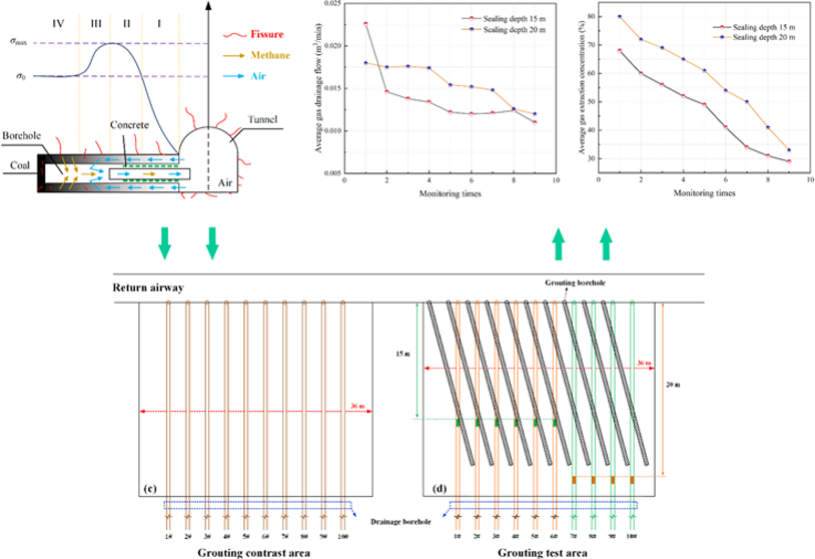

Drilling for gas extraction is widely used as the main approach to manage gas in mines. However, gas leakage during borehole extraction reduces the root cause of the effectiveness of gas extraction. Given that forming a normal hole in the prominent coal seam of the Qingdong Coal Mine is impossible and that air leakage leads to difficulties in prepumping gas in the coal seam, we selected coal seam 3# as the object of this study. First, qualitative analysis determined that the air leakage channel restricted effective gas extraction. Second, short-hole grouting and plugging were proposed to increase the concentration and efficiency of gas extraction from the coal wall, forming a barrier by blocking the fissure network in the plastic zone of the surrounding rock of the coal roadway and preventing the air inside the roadway from penetrating into the coal seam and gas extraction drill holes. Finally, evaluation of the gas extraction efficiency between the grouting test and comparison areas indicated that the initial gas extraction concentration of a single hole could reach 89% when the depth of the selected blocking hole was 15 m. Grouting slowly decreased the gas extraction concentration from 72 to 25%, which effectively improved the speed at which the gas content was reduced in the coal body. The study findings provide a field basis for similar mines to improve their gas extraction efficiency and extend their extraction time.

## 1 Introduction

China’s coal resources are rich and widely distributed. As an important natural resource, coal occupies a dominant position in China’s energy structure.^1,2^ Nevertheless, with increasing coal mining depth, which yields increased gas content and increases the outflow of coal seams, the probability of gas disasters gradually increases.^3^ Coal mine methane (CMM), the main component of which is methane gas, is not only a highly efficient energy source but also a natural factor that seriously threatens the safe production of coal mines. Safety hazards such as gas explosions and coal gas outbursts can cause large numbers of casualties and huge property losses.^4,5^ Presently, gas extraction by drilling is an important approach to control CMM,^6^ which is mainly achieved by drilling extraction holes from the roadway to the coal seam and utilizing the negative pressure to extract the gas inside the coal seam, which in turn reduces the risk of gas disaster.

Drilling holes to extract gas is affected by factors such as coal seam permeability, gas adsorption capacity, and gas pressure, resulting in low extraction efficiency and other problems.^7^ At present, the quality of approximately 2/3 of the gas mines in China does not meet gas extraction requirements.^8^ The root cause of the poor gas extraction efficiency is gas leakage during borehole extraction. Coal seam drilling disturbs the stress balance between coal and rock bodies around the drill hole, causing the primary pores and fissures inside the coal body to gradually expand and a large number of secondary fissures to develop. This results in a sharp increase in the permeability coefficient of the coal body in the drilling range, forming an air leakage channel from the drill hole. Under the effect of negative pressure, a large amount of outside air can flow into the borehole through the air leakage channel, which greatly decreases the gas concentration during late-stage borehole extraction, reduces the negative pressure of extraction, and causes a rapid decline in the amount of pure gas extracted.^9−11^ Scholars have conducted substantial engineering research on drill hole sealing materials to improve the concentration and efficiency of gas extraction.^12−14^ Common sealing materials are clay-, polyurethane-, and cement-based materials. Clay is inexpensive and includes a wide range of materials; however, achieving efficient borehole degassing with the clay sealing method, as a manual operation, is time-consuming and labor-intensive.^15^ Although convenient, polyurethane is not effective in sealing drill holes due to its irritating odor and the fact that it can only fill large fissures near the well wall.^16^ Meanwhile, cementitious materials are widely used owing to their good fluidity and supportive nature.^17,18^

To achieve efficient gas drainage from boreholes, Hu et al. proposed a particular bag-type borehole sealing device.^19^ Fu et al. developed a material with dual expansion sources to seal boreholes via bag grouting sealing, which improved gas drainage efficiency in boreholes.^20^ Liu et al. established a full-force composite flow model to reveal the air leakage mechanism around the borehole, which they combined with cement grouting and gel injection to optimize borehole sealing and effectively improve the quality of borehole gas extraction.^4^ Additionally, a large number of studies have proposed various efficient drill hole sealing techniques, achieving good results. Zhang et al.^21^ analyzed the factors influencing air leakage in drilling via simulation, constructed a model of air leakage in drilling, and proposed an integrated sealing–isolation technique, which improved gas extraction efficiency compared with the traditional sealing technique. Similarly, Liu et al.^22^ revealed three shortcomings of the traditional sealing method and proposed an integrated sealing technique with grouting twice or more under high grouting pressure, achieving good results by using a 1:0.8 cement slurry ratio as the sealing material. Furthermore, Li et al.^8^ proposed an integrated drilling, protection, and sealing technology for drilling holes in view of the geological characteristics of soft coal seams, utilizing a new type of airbag grouting technology to ensure the sealing of the drill holes. Xiang et al.^23^ proposed a new sealing method using a flexible gel sealing material as the grouting material, which utilized the advantages of a flexible gel material to realize active borehole sealing, thereby improving gas extraction efficiency.

However, the existing hole sealing process often ignores the development of fissures in the surrounding rock of the roadway caused by coal mine excavation. Therefore, this paper focuses on the problem that holes cannot be properly formed in the prominent coal seam in the Qingdong Coal Mine and air leakage leads to difficulties in prepumping gas in the coal seam. Herein, we qualitatively analyze the mechanism of air leakage during drilling and extraction, verify the accuracy of the mechanism based on measured data at the site, and propose short-hole grouting and plugging of the coal wall of the roadway gang to control air leakage from the main channel in order to enhance the gas extraction efficiency. We then apply this sealing approach to the site to verify the method’s feasibility and obtain the optimal depth of sealing holes. The study findings have engineering practice significance for improving drilling and mining efficiency and the safe recovery of the working face.

## 2 Overview of the Working Face

**Figure 1:**
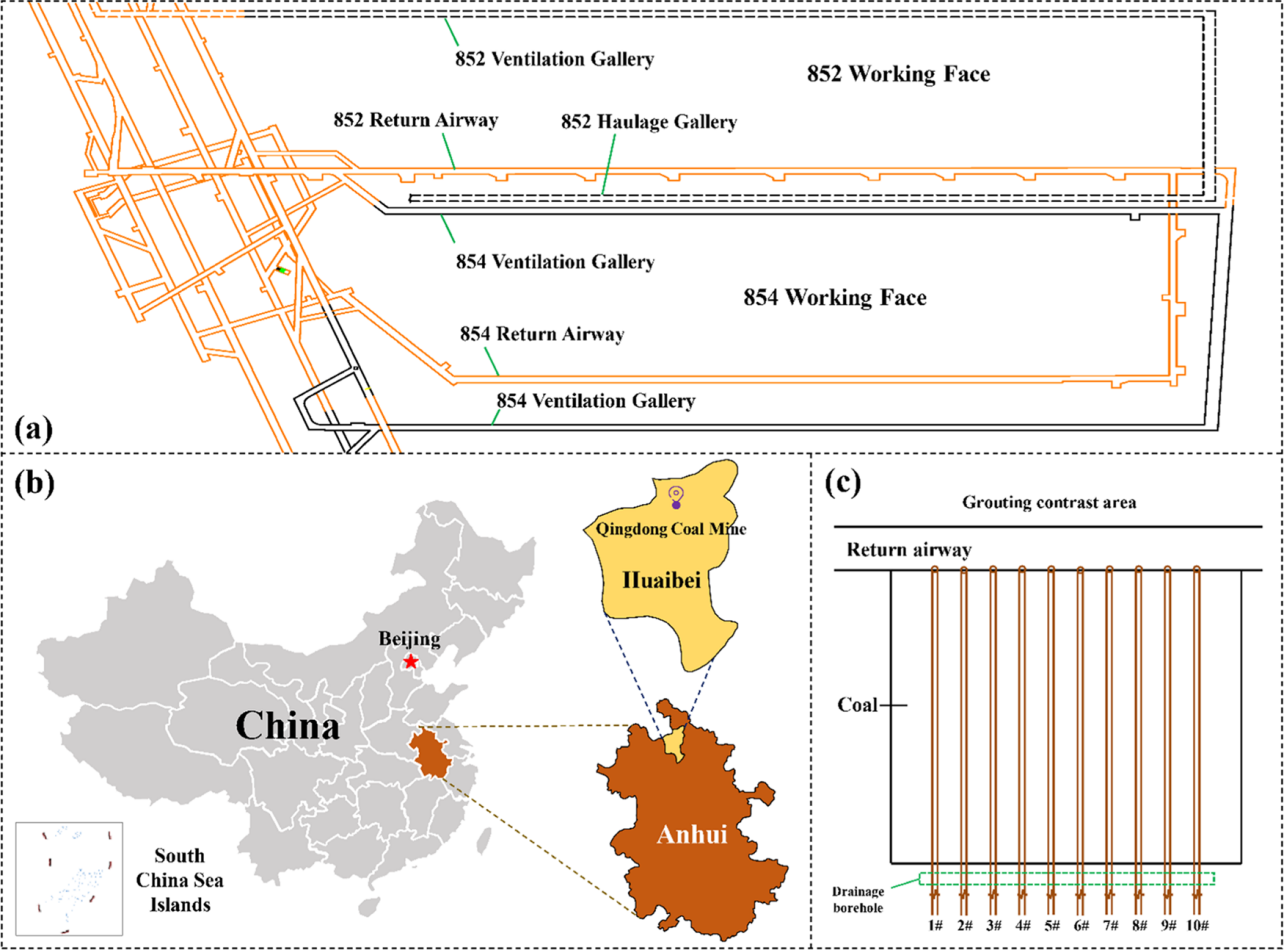
Schematic location of the Qingdong Coal Mine and working face 854. (a) Schematic diagram of working face 854. (b) Location of the coal mine.^24^ (c) Drill hole arrangement. Adapted or reprinted in part with permission from ref (24). Copyright [2018] [MDPI/Wang, G.].

The Qingdong Coal Mine is located in Huaibei City, Anhui Province, China, neighboring the Cuilou Coal Mine in the north and the Hazi and Linhuan Coal Mines in the east. The specific location is shown in Figure 1. Working face 854 was selected as the research object: the thickness of the working face is 7.76–17.34 m; the inclination angle of the coal seam is 2–21°, with an average of approximately 10°, and the average coal thickness is 11.09 m. The mining and release ratio is 1:3.32. The machine lane is the inlet lane, the wind lane is the return lane, the difference in elevation is approximately 58.4 m, and the horizontal distance is 160 m. Real gas pressure measurements were taken in 8 coal seams, in which working face 854 is located. The maximum original gas pressure was 0.6 MPa (elevation: −642 m), the maximum original gas content was 5.71 m^3^/t (elevation −625 m), and the expected gas outflow of the 854 working face was 5.256 m^3^/t. Carrying out intensive extraction measures for working face 854 is needed to further reduce the gas content in the wind flow before mining.

However, forming holes is often difficult when drilling in the Qingdong Coal Mine, which prevents using a conventional hole sealing process to effectively extract gas in the coal seam. Therefore, a short-hole sealing process was proposed, i.e., arranging a short hole to be grouted in the periphery of the extraction drill hole in advance. This process forms a blocking barrier in the unloading area of the coal seam, which reduces the degree of fragmentation in the unloading area of the coal seam, prevents air leakage from the coal wall, increases the gas concentration in the extraction borehole, slows the decay of the gas extraction rate, and increases the amount of gas extracted from a single borehole, thus realizing sustained, efficient, and safe mining of the working face. Meanwhile, after the grouting material is cemented and cured within the coal body, the mechanical strength of the coal body within the grouting area is improved, which is conducive to improving the hole formation rate during downstream drilling for prepumping gas.

## 3 Analysis of the Attenuation Pattern of Wind Leakage from Drilling and Extraction

### 3.1 Mechanism of Air Leakage from Drilling and Extraction

In the process of gas extraction, the air outside the coal body is constantly mixed into the extracted gas flow through the fissure channel of the surrounding rock in the coal roadway, which reduces the gas concentration. The fissures in the coal wall of the roadway, secondary fissures in drilled holes, incompact voids in the sealing section, and fissures in the coal seam caused by mining constitute the main air leakage channel in extraction drilling are the main reasons for reduced gas extraction efficiency.^25−27^

#### 3.1.1 Wind Leakage from the Coal Wall Side of the Roadway

**Figure 2:**
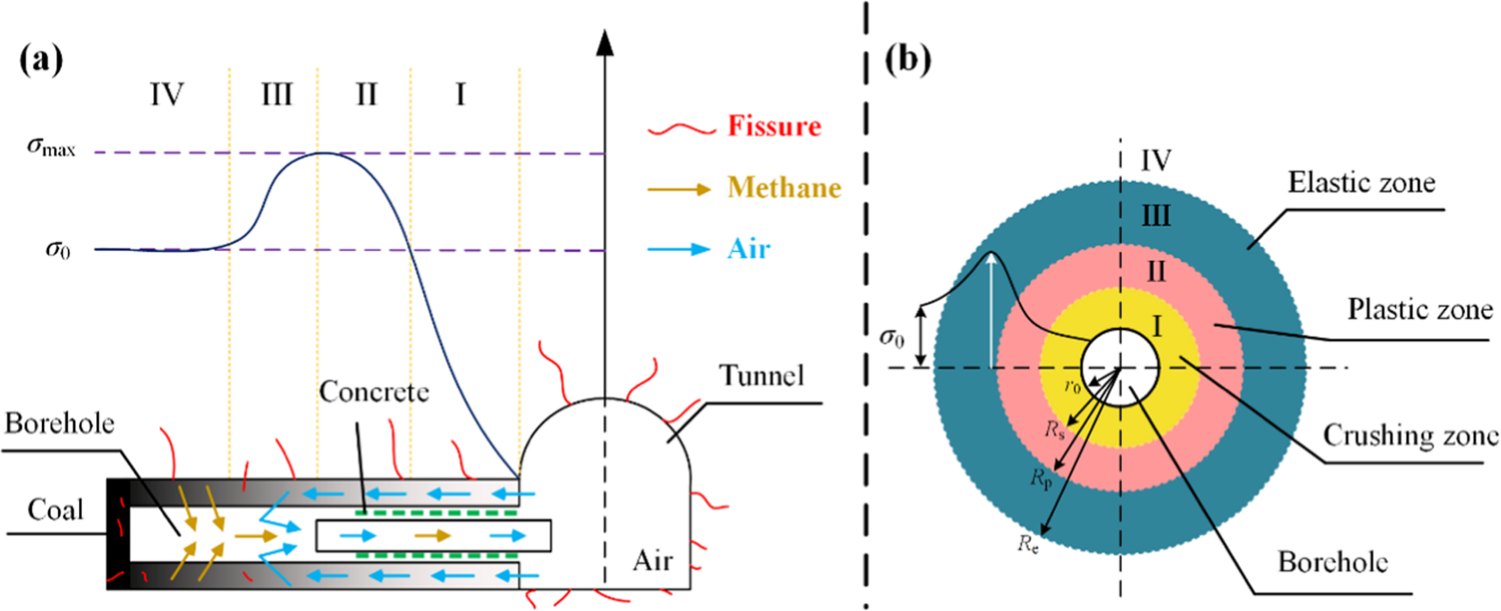
Schematic diagram of the stress distribution pattern of the coal body around the roadway and drill hole. (a) Stress distribution of the coal body in the roadway. (b) Stress distribution around the drill hole.

During roadway excavation, the original long-term stable equilibrium between coal seams and the rock layer is destroyed, and the coal body on the side of the coal wall at the head of the excavation fracture is broken.^28,29^ Thus, the stress series of new fissures along the roadway direction, the coal seam, and the rock layer need time after the excavation process to regain equilibrium. At this time, the state of the coal in the coal body can be divided into stress drop and stress rise zones. In this case, the stress state of the coal body can be divided into stress decrease, stress increase, and original rock stress zones, as shown in Figure 2(a). The coal body in the stress drop zone is most seriously affected by mining work, and a large number of macroscopic fissures are generated within. Permeability is greatly improved, and the primary fissures in the coal body are connected to become the main channel for gas leakage from drill holes.^30,31^ The stress rise and original rock stress zones are less affected by the mining work.

#### 3.1.2 Air Leakage from Secondary Fissures around Drill Holes

Drilling also causes stress changes in the coal body around the drill hole, similar to the stress changes in the coal body caused by roadway excavation,^32,33^ as shown in Figure 2(b). Taking the borehole as the center, the coal body can be divided into stress reduction, stress increase, and original rock stress zones from the inside to the outside, and the main air leakage area from the borehole is the stress reduction zone, which is distributed in a ring shape along the vertical direction of the borehole. The coal body is deformed and broken when the peak stress is higher than the critical strength of the coal, resulting in the generation of a large number of fissures and formation of a secondary fissure network around the borehole, which provides a flow channel for air leakage from the borehole.

#### 3.1.3 Air Leakage from the Void in the Sealing Section of the Drill Hole

Air leakage in the sealing section of the drill hole is mainly caused by the air leakage channel formed by cracks in the coal body.^34−36^ Issues such as the gap between the sealing material and the wall of the drill hole, pores in the sealing material caused by construction of the drill hole, and the air leakage channel formed by the wall of the drill hole and sealing material have been adequately solved through continuous improvements in understanding the air leakage mechanism of the drill hole.

### 3.2 Calculation of Wind Leakage Attenuation Law in Drilling and Extraction

The gas concentration in the initial extraction state of the borehole is high, with most of the gas nearest to the borehole extracted with increased extraction time in the initial stage, and then the radius of influence of extraction continues to expand, and gas begins to be extracted further away. During this time, the borehole interacts with more fissure passages and the amount of air leakage increases, resulting in a gradual decrease in gas concentration with extraction time. Therefore, analyzing the gas concentration change in the drill holes of this coal seam and calculating the air leakage volume according to the gas flow rate can help to elucidate changes in drill hole air leakage patterns under normal conditions in the Qingdong Coal Mine.

#### 3.2.1 Gas Extraction Volume

The amount of gas extracted from drill holes decays with time, as given by the following relationship
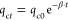
1where *q*_c0_ is the initial gas extraction volume of the borehole, m^3^/(min·hm); *q*_c*t*_ is the average gas extraction volume of the borehole at extraction time *t*, m^3^/(min·hm); β is the decay coefficient of the gas extraction volume of the borehole, d^–1^; and *t* is the gas extraction time of the borehole, d.

#### 3.2.2 Air Leakage during Extraction

Under the conditions of relatively stable negative pressure during borehole extraction, the mixed flow rate *q* of gas from the extraction borehole is certain, and the air leakage in the extraction mix is given by
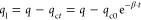
2where *q*_l_ is the air leakage at a certain moment, m^3^/(min·hm).

### 3.3 Field Analysis of the Attenuation Law in Drilling and Extraction

Two extraction drill holes (i.e., boreholes 9 # and 10 #) in the grouting comparison area adjacent to the short-hole grouting test area were selected to analyze gas concentration changes in the extraction boreholes of this coal seam, and the air leakage amount was calculated according to the gas flow rate to obtain the changes in borehole air leakage patterns under normal conditions in the Qingdong Coal Mine.

**Figure 3:**
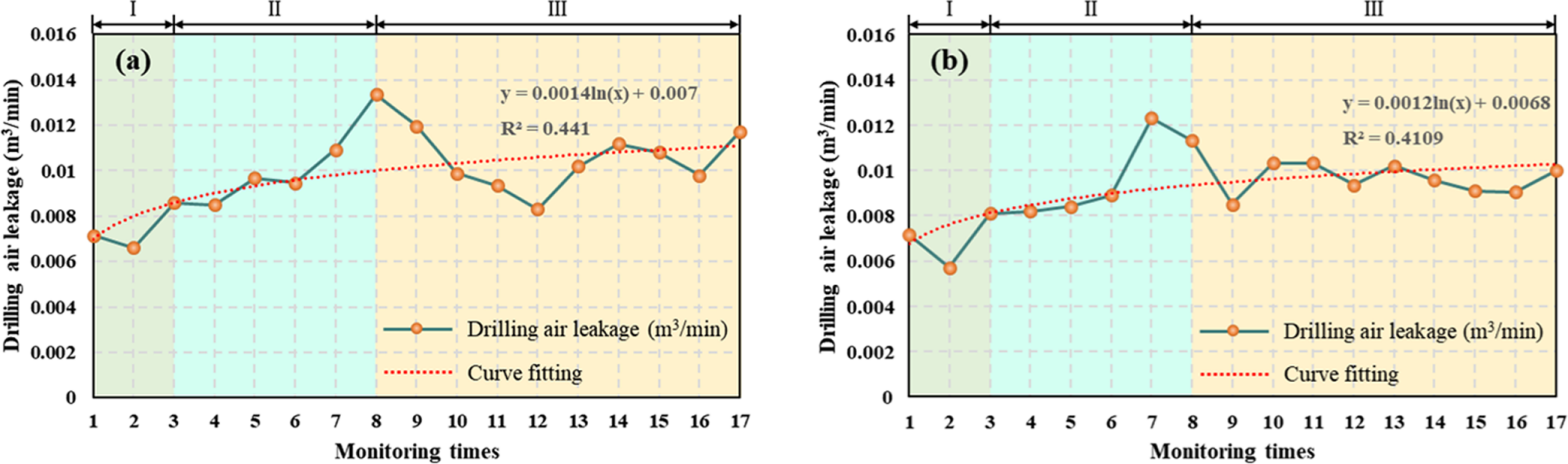
Borehole air leakage map. (a) Air leakage extraction study for hole 9#; (b) Air leakage extraction study for hole 10#.

Monitoring of this extraction borehole was divided into three stages. Stage I comprised the first and second monitoring times on days 1 and 2 of monitoring, respectively, totaling 2 days. Stage II started from the third monitoring time, with 7 days of monitoring, until the beginning of the eighth monitoring time, totaling 35 days. Stage III started from the eighth monitoring time, with 15 days of monitoring, until the end of the 17th monitoring time, totaling 150 days. In total, there were 17 sets of data for 187 days. The details are listed in Figure 3.

According to Figure 3, the fitting degrees of the air leakage curves for extraction holes #1 and #2 were 0.441 and 0.4109, respectively. Although the fitting degree of the air leakage curves of the two holes was low, the trend in the air leakage of the holes is reflected by the curve. The air leakage volume gradually increased with pumping time, and the increase was small. The increasing pattern of air leakage approximately conformed to a logarithmic function, which was mainly attributed to gas outflow in the air leakage channel at the beginning of gas extraction and hindered air leakage. With extended extraction time, the air leakage channel gradually opened, and the air leakage volume increased and eventually stabilized at a constant value, which was consistent with air leakage theory. However, air leakage did not infinitely increase; the radius of influence of extraction under negative pressure did not further increase after a certain extraction time, and the increase in air leakage gradually stabilized. At this time, the amount of pure gas extracted could only be increased by increasing the negative pressure of extraction, achieved by increasing the pressure difference between the inside and outside of the coal, which would also cause an increase in the extraction load.

## 4 Coal Bed Short-Hole Grouting and Thickening Process

### 4.1 Short-Hole Grouting Process Design

**Figure 4:**
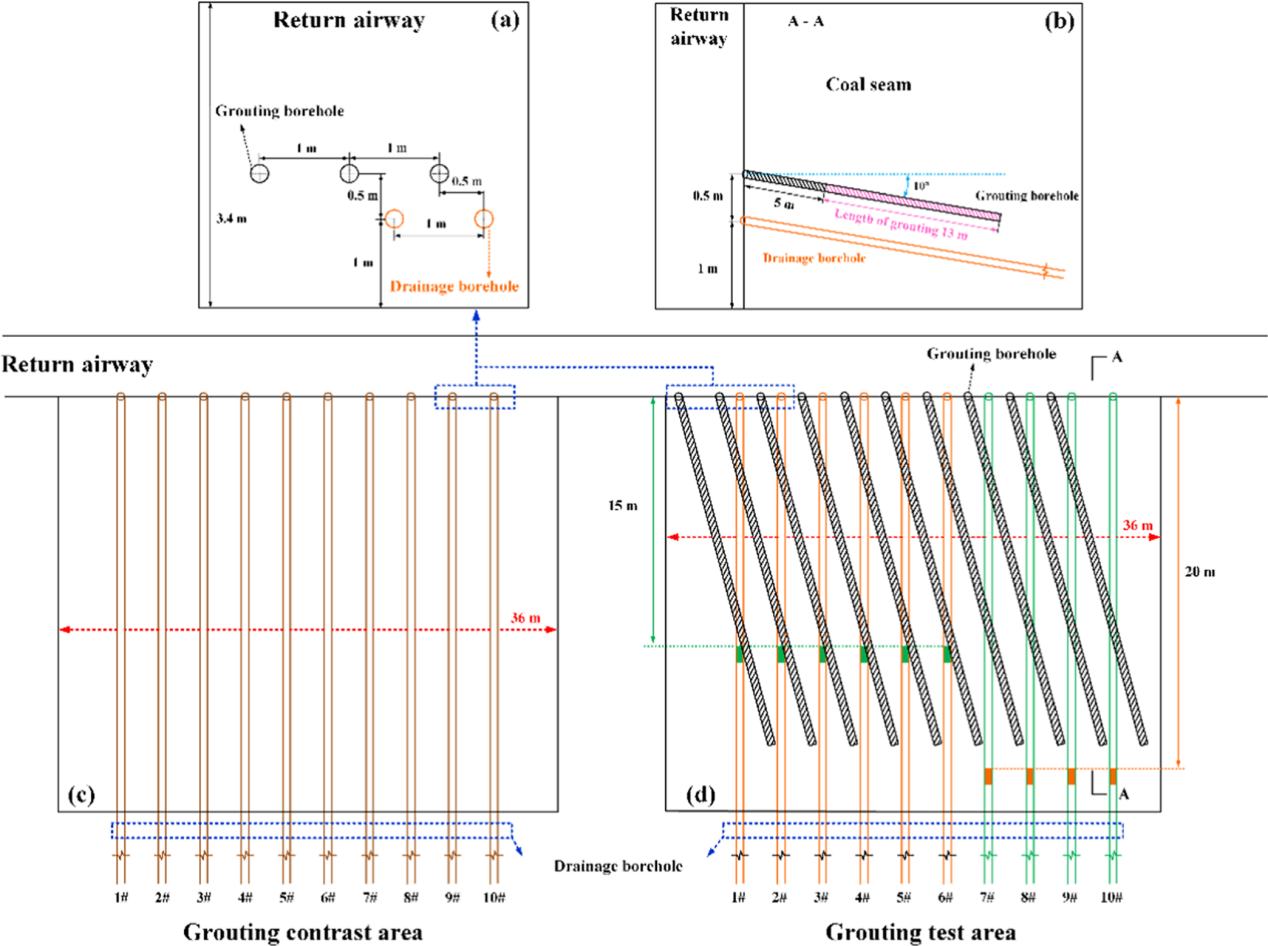
Schematic diagram of short-hole grouting and extraction drilling. (a) Drill hole opening location. (b) Grouting and extraction hole profile. (c) Grouting comparison area. (d) Grouting test area.

The principle of short-hole grouting is to prevent air leakage from the angle formed by the air leakage channel between the drilling hole and the coal wall by constructing short holes at certain depths in the roadway gang and injecting a blocking liquid with good diffusivity. On the one hand, the blocking liquid can block the fissure channel in the coal seam and prevent air leakage to the drilling hole; on the other hand, it can improve the strength of the coal body and reduce the degree of crushing. The length of the test area for this short-hole grouting and thickening process was approximately 36 m. The ungrouted comparison area was set up in the same roadway, and the specific location is shown in Figure 4(a).

To realize the maximum range of slurry diffusion while arranging the minimum number of grouting holes in the grouting test area, the diffusion radius (0.9 m) of the grouting liquid was determined through a slurry seepage diffusion radius test prior to grouting. Then, 10 short grouting holes were designed in the vertical coal wall at a distance of 1 m from the first short hole with a spacing of 2 m. The opening height of the short holes was 1.5 m; the inclination angle of the holes was −10°, the depth of the drilling holes was 18 m, and the horizontal projected length was 17.7 m. The specific hole arrangement is shown in Figure 4(b). After construction of the short grouting holes was complete, the cement slurry material (JD-WFK-2) was used for grouting at a pressure ≥1.2 MPa (the orifice pipe or grouting pump was equipped with a pressure gauge to show the grouting pressure). All holes were sealed by pressure grouting, and the outer side of the sealed holes was 5 m from the mouth of the holes. To investigate the effect of different sealing depths on the gas extraction efficiency after grouting, the 10 extraction holes were divided into two groups: the first 6 extraction holes (1#–6#) were sealed at a depth of 15 m, and the last 4 extraction holes (7#–10#) were sealed at a depth of 20 m. The ungrouted comparison area was designed for the construction of only 10 gas extraction holes in the downstream layer with a sealing depth of 20 m, and the rest of the parameters were the same as those of the grouting test area.

### 4.2 Field Effectiveness Testing

#### 4.2.1 Comparison of the Pumping Effect between Grouted and Ungrouted Areas

**Figure 5:**
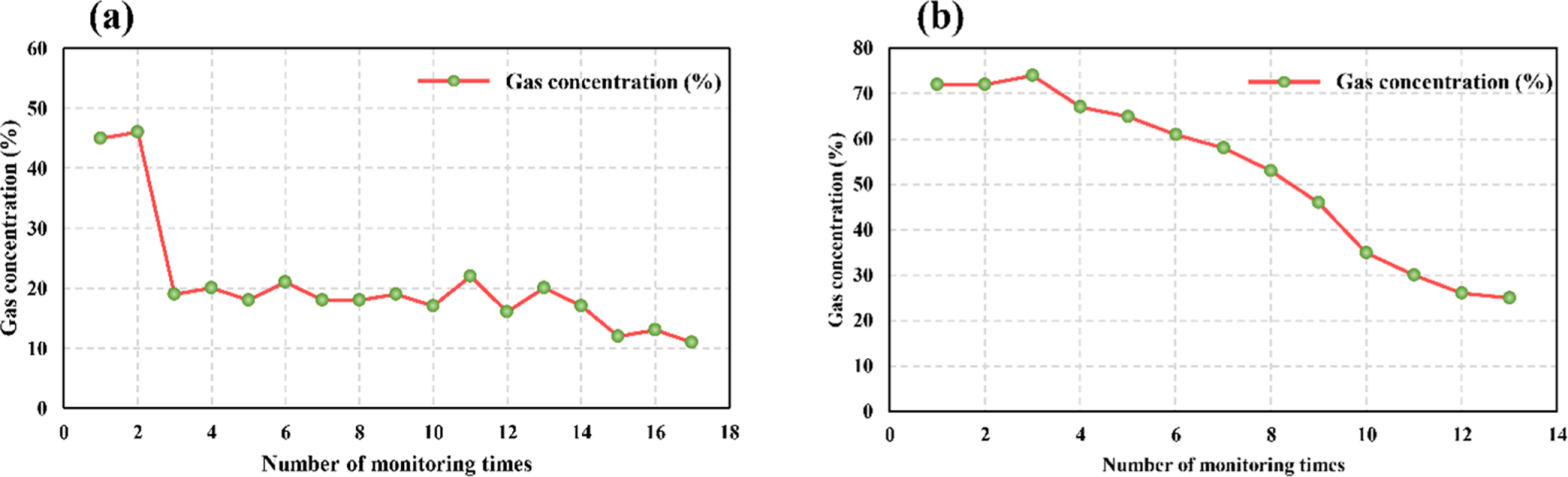
Comparison of the fitted curves of gas extraction concentration in the grouting test and comparison areas: (a) grouting comparison area and (b) grouting test area.

The conditions and monitoring cycles of the coal seams in the grouting test and comparison areas were basically similar; therefore, the data from the extraction boreholes in the two areas could be compared and analyzed. Figure 5 shows a comparison of the fitted curves of the gas extraction concentration in the grouting test and comparison areas.

As shown in Figure 5(a), the gas extraction concentration of the borehole in the grouting test area rapidly decreased to ≤20% after 1 week of extraction, and then the gas concentration was maintained at approximately 20% during the 6 month monitoring cycle with a peak gas extraction concentration of 46%, and a minimum of 11%. As shown in Figure 5(b), the gas extraction concentration of the borehole in the grouting test area was always >20%, with an initial concentration of 72% and a peak concentration of 74%. The gas concentration slowly decreased in the first 2 months of the monitoring period from 72 to 58%, with a decrease of only 14%.

**Figure 6:**
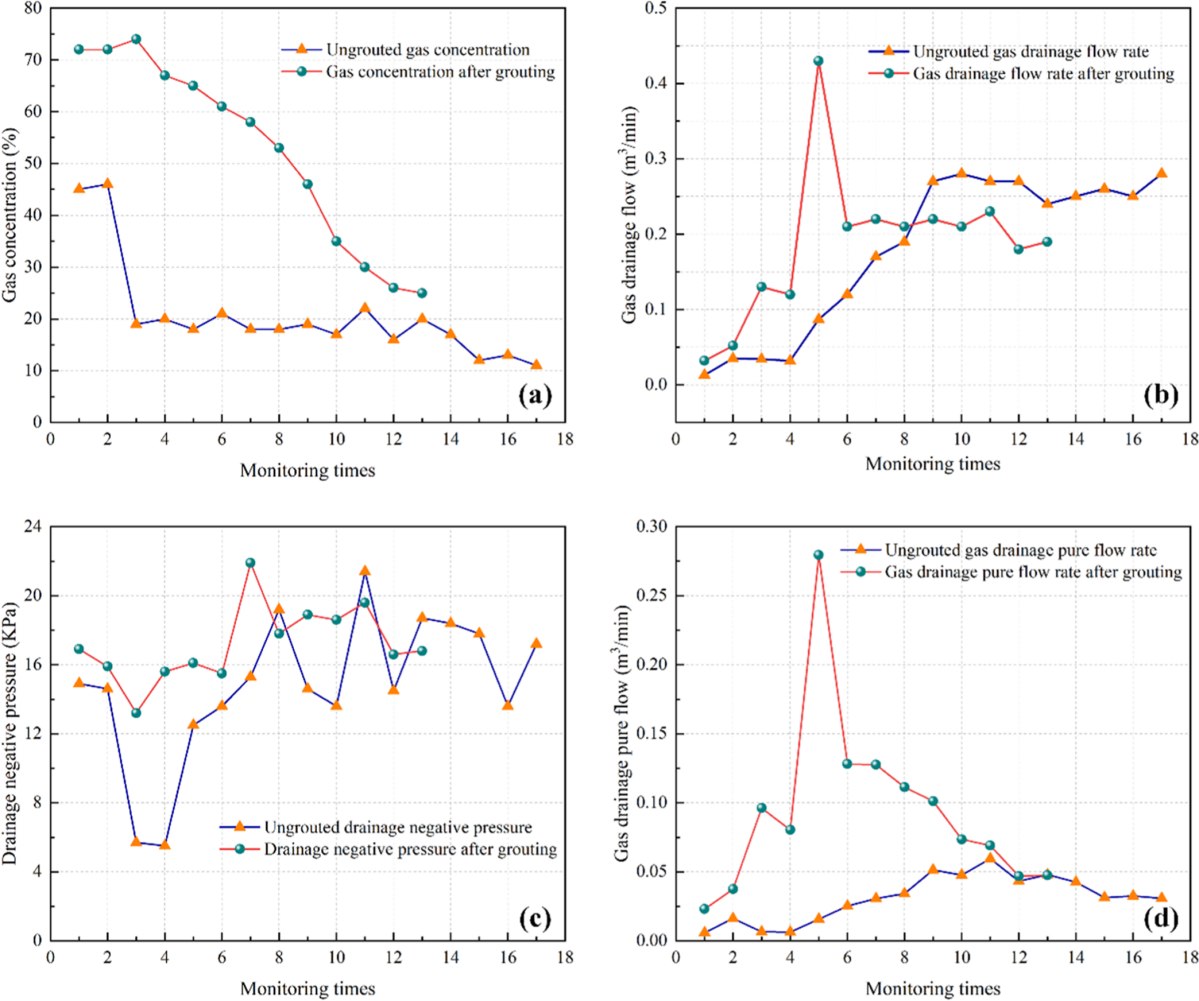
Comparison of gas extraction concentration, mixed extraction flow rate, negative gas extraction pressure, and amount of pure gas extracted between the grouting test and comparison areas. (a) Gas extraction concentration. (b) Gas mixed extraction flow rate. (c) Negative gas extraction pressure. (d) Amount of pure gas extracted.

Comparisons of the gas extraction concentration, mixed extraction flow rate, negative gas extraction pressure, and amount of pure gas extracted between the grouting test and comparison areas are shown in Figure 6. As shown in Figure 6(a), the difference between the peak gas extraction concentration before and after grouting was 28%, and the gas concentration in the grouting test area was reduced from 72 to 25%, which indicated that the short-hole grouting plugging measure effectively improved the gas extraction concentration of the downstream gas extraction borehole. Furthermore, it took 7 days for the gas concentration to decrease to 20% without grouting and compared to 187 days for the gas concentration to decrease to 25% with grouting, indicating that the short-hole grouting and plugging measure reduced the decay of the gas extraction rate, increased the effective extraction time of the boreholes, and effectively reduced the residual gas content in the coal seam.

Figure 6(b) displays the data from the seventh monitoring time: the gas extraction flow rate in the grouting comparison area was 0.17 m^3^/min, while that in the grouting test area was 0.22 m^3^/min, with a difference of only 0.05 m^3^/min. However, the gas concentration in the grouting comparison area was 18%, while that in the grouting test area was 58%, with a difference of 30%. This finding indicated that in extraction holes with small differences in the gas extraction flow rate, the gas concentration of extraction holes plugged with grout was higher than that of ungrouted holes, indicating that short-hole grouting had a positive effect on the gas concentration of extraction boreholes.

As shown in Figure 6(c,d), the average negative pumping pressure of the boreholes in the grouting comparison area (14.77 KPa) was smaller than that of the boreholes in the grouting test area (17.18 KPa), with a difference of 2.41 KPa, which indicated that short-hole grouting played an important role in blocking the fissures around the inner part of the boreholes. Additionally, the peak volume of pure gas extracted in the grouting test area (0.069 m^3^/min) was larger than that in the grouting comparison area (0.059 m^3^/min), with a difference of 0.01 m^3^/min. Therefore, the short-hole grouting and plugging measures effectively increased the gas extraction efficiency and concentration of the extraction drill hole. Grouting played a vital role in thickening the gas extraction boreholes in the downstream layer.

#### 4.2.2 Analysis of the Gas Concentration and Flow Rate of Extraction Boreholes

**Figure 7:**
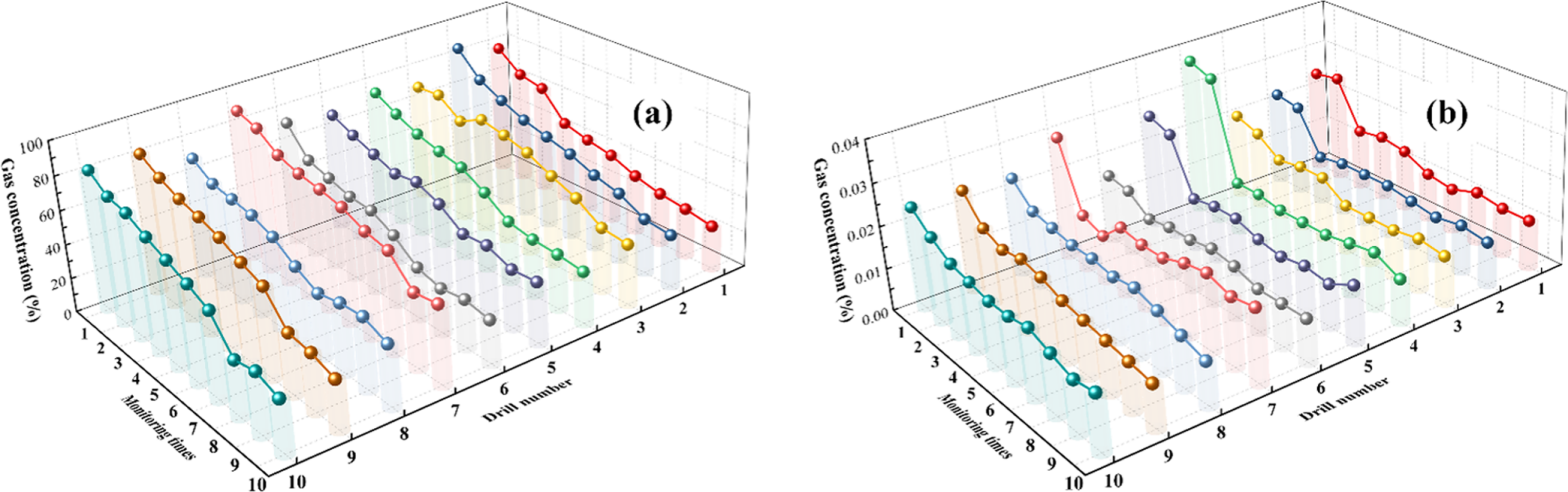
Integration diagram of gas concentration and flow rate of extraction boreholes. (a) Gas extraction concentration. (b) Volume of pure gas extracted.

In order to better observe the attenuation pattern of gas extraction boreholes and analyze the interrelationship between different extraction boreholes, the 10 boreholes in the grouting test area were integrated, and 10 monitoring times were selected for approximately 3 months, as shown in Figure 7.

As shown in Figure 7(a), the initial gas concentration of the 10 extraction boreholes in the grouting test area was >70%, with the initial gas concentration of extraction boreholes 2#, 7#, 9#, and 10# reaching >80%. The gas extraction concentration remained >20% at the end of the monitoring period. The change in the gas extraction flow rate followed the same decreasing trend as the change in gas extraction concentration but with a relatively small change in magnitude. Therefore, when analyzing the gas extraction concentration and flow rate of the extraction boreholes from an overall perspective, the increase in gas extraction concentration was most obvious in the short-hole grouting test area.

#### 4.2.3 Comparative Analysis of the Extraction Efficiency of Different Sealing Depths

**Figure 8:**
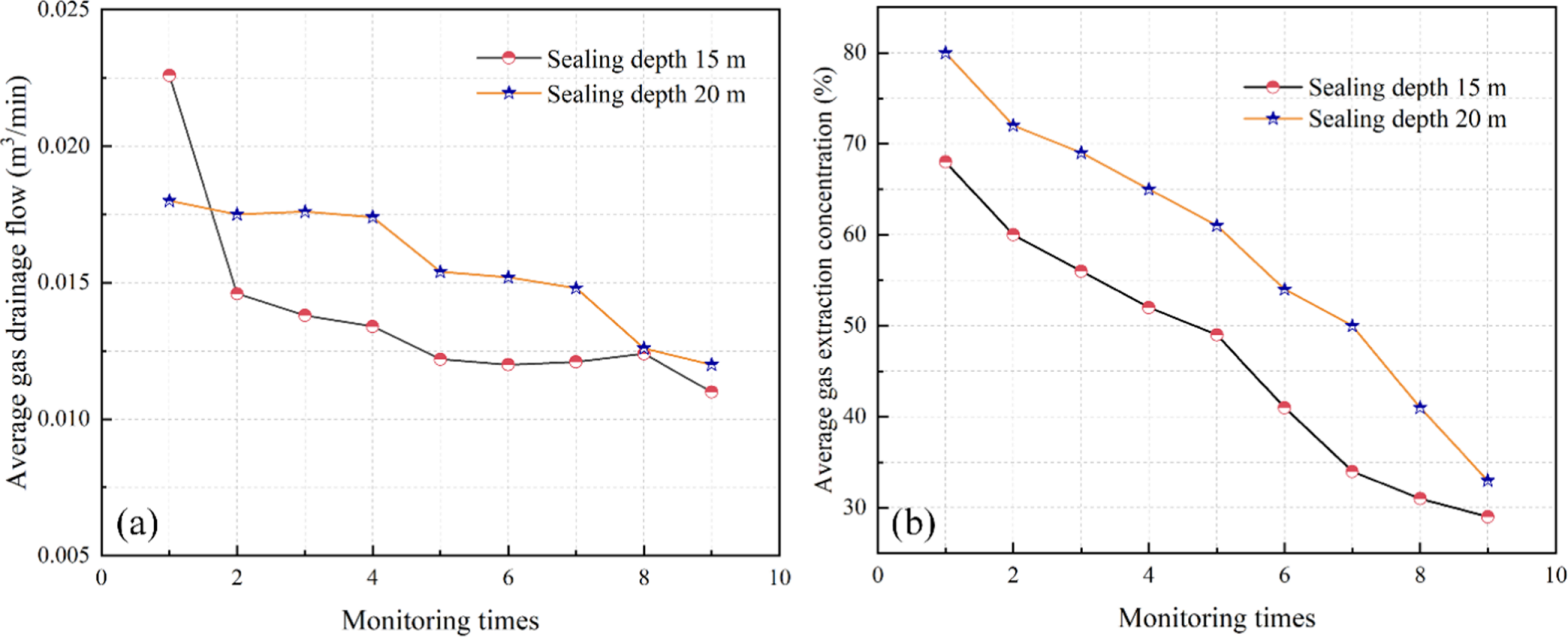
Comparison of the effect of extraction boreholes with different sealing depths in the same observation period. (a) Average gas drainage flow. (b) Average gas drainage concentration.

In order to facilitate the analysis of the reasonable sealing depth of extraction boreholes for grouting, data starting from day 67 (with monitoring number 10) were selected to compare and analyze the average gas extraction concentrations and flow rates of extraction boreholes 1–6# and 7–10# in the grouting test area, as shown in Figure 8.

As shown in Figure 8, after adopting the short-hole grouting sealing measure in the grouting test area, the average gas extraction flow rate and concentration were smaller at a sealing depth of 15 m than at a sealing depth of 20 m; the difference was relatively small, and they maintained the same decreasing trend. Starting from day 67 (monitoring number 10), the average gas extraction flow rate and concentration of the extraction hole with a sealing depth of 15 m continued to stabilize at approximately 0.11 m^3^/min and 30%, respectively, while the average gas extraction flow rate and concentration of the extraction hole with a sealing depth of 20 m continued to steadily decrease. Therefore, after the short-hole grouting sealing measure was adopted, a sealing depth of 15 m achieved better gas extraction efficiency than a sealing depth of 20 m.

## 5 Discussion

Gas in the coal body mainly consists of free gas in the fissures and adsorbed gas in the coal matrix. Although adsorbed gas accounts for a larger proportion, free gas in the fissures can be extracted under the effect of negative pressure.^37^ Therefore, in the process of gas extraction in the downstream layer, the transportation of air and free gas in the fissures and boreholes obeys Darcy’s law, i.e., the driving force of transportation is pressure.^38^

When coal roadway excavation is complete, the surrounding rock of the roadway has areas with different ranges of unpressurization due to the buried depth of the coal seam and the cross-sectional area of the roadway, resulting in the fissure pressure in the unpressurized area of the coal seam being the same as the atmospheric pressure, which is approximately 0.1 MPa. Under the action of the fan’s pressure supply, the air in the coal roadway penetrates deeper into the coal seam through the fissures in the surface of the coal wall of the surrounding rock of the roadway. Thus, the fissures in the unpressurized area of the coal seam are already filled with infiltrated air before the construction of the gas extraction boreholes.

When gas extraction drilling is underway, the fissures in the coal seam surrounding the drilled rock are also damaged, producing the unloading zone, and at this time, the air continues to infiltrate into the unloading zone of the coal seam through the coal wall and drilled hole. When the gas pressure in the fissures surrounding the drill hole is lower than the air pressure in the fissures surrounding the roadway, air will be driven by the pressure gradient into the fissures of the coal seam and transported along the fissure network in the rock surrounding the drill hole. At the same time, drilling damages the coal matrix, and gas is continuously desorbed and released, mixing with air in the fissures of the perimeter rock of the borehole, flowing into the interior of the borehole, and then leaving the coal seam under the action of negative pressure of extraction.^39,40^ However, according to the drilling data of different sealing depths and areas in the field, the extracted gas always contains air, which greatly affects the gas extraction efficiency. Therefore, taking the source of air infiltration into the coal seam as the starting point, we proposed a short-hole grouting and sealing measure to block the fissures in the unloading zone of the coal seam in the surrounding rock of the roadway, effectively blocking air infiltration from the source. This measure improved the extraction efficiency of downstream gas extraction drilling, increased the gas extraction concentration from a single hole, and lengthened the effective extraction time.

## 6 Conclusions

(1) Combined with the air leakage theory from the coal wall, we qualitatively analyzed the stress equilibrium state of the surrounding rock damaged by roadway excavation. Our findings indicated that under the action of wind pressure in the roadway, the air in the roadway penetrates into the plastic damage zone through the fissures in the surface of the coal wall, mixes with free gas in the network of fissures in the coal wall, and then leaves the coal seam under the action of the negative pressure of extraction. In this process, the gas extraction concentration in the gas extraction drilling holes is reduced due to mixing with air, resulting in a high gas concentration accumulating in the coal seam, which is a great safety hazard for mining operations.
(2) The air leakage channel has a restraining effect on gas extraction efficiency. According to the data obtained from two extraction holes in the short-hole grouting test area adjacent to the grouting comparison area, the attenuation law of gas extraction is in the form of a logarithmic function. The air leakage channel, which is hindered by the influence of gas outflow at the beginning of extraction, gradually opens with extended extraction time, increasing the air leakage volume, which gradually stabilizes.
(3) Short-hole grouting was used to seal the fissures in the plastic zone of the surrounding rock of the coal tunnel, which formed a barrier from the source in the area, blocking the air inside the roadway from infiltrating into the coal seam and the gas extraction drill holes. The sealing depth of the gas extraction drilling holes in the grouting test area was selected as 15 m, and the initial gas extraction concentration of a single hole reached up to 89%, which was slowly reduced from 72 to 25%. These results fully demonstrated that the short-hole grouting blocking measure could effectively improve the speed at which the gas content in the coal body was reduced and greatly prolong the duration of extraction, playing a positive role in ensuring safe mining.

## References

[ref1] Wang Z.; Sun Y.; Li Z.; Wang Y.; You Z. Multiphysics responses of coal seam gas extraction with borehole sealed by active support sealing method and its applications. J. Nat. Gas Sci. Eng. 2022, 100, 104466 10.1016/j.jngse.2022.104466.

[ref2] Wang H.; Fan C.; Li J.; Zhang Y.; Sun X.; Xing S. Dynamic characteristics of near-surface spontaneous combustion gas flux and its response to meteorological and soil factors in coal fire area. Environ. Res. 2023, 217, 114817 10.1016/j.envres.2022.114817. 36395860

[ref3] Fan C.; Xu H.; Wang G.; Wang J.; Liu Z.; Cheng Q. Determination of roof horizontal long drilling hole layout layer by dynamic porosity evolution law of coal and rock. Powder Technol. 2021, 394, 970–985. 10.1016/j.powtec.2021.09.002.

[ref4] Liu T.; Lin B.; Fu X.; Zhu C. Modeling air leakage around gas extraction boreholes in mining-disturbed coal seams. Process Saf. Environ. Prot. 2020, 141, 202–214. 10.1016/j.psep.2020.05.037.

[ref5] Zheng C.; Chen Z.; Kizil M.; Aminossadati S.; Zou Q.; Gao P. Characterisation of mechanics and flow fields around in-seam methane gas drainage borehole for preventing ventilation air leakage: A case study. Int. J. Coal Geol. 2016, 162, 123–138. 10.1016/j.coal.2016.06.008.

[ref6] Junxiang Z.; Bo L.; Yuning S. Dynamic leakage mechanism of gas drainage borehole and engineering application. Int. J. Min. Sci. Technol. 2018, 28 (3), 505–512. 10.1016/j.ijmst.2018.04.008.

[ref7] Kang Y.; Geng Z.; Liu B.; Chen J. Study on gas extraction efficiency using in-seam borehole method considering influence of plastic zone induced by borehole drilling. Geomech. Energy Environ. 2023, 33, 100426 10.1016/j.gete.2022.100426.

[ref8] Li H.; Wang W.; Liu Y.; Ma J.; Gao H. An integrated drilling, protection and sealing technology for improving the gas drainage effect in soft coal seams. Energy Rep. 2020, 6, 2030–2043. 10.1016/j.egyr.2020.07.017.

[ref9] Cai J.; Wu J.; Yuan S.; Liu Z.; Kong D. Numerical analysis of multi-factors effects on the leakage and gas diffusion of gas drainage pipeline in underground coal mines. Process Saf. Environ. Prot. 2021, 151, 166–181. 10.1016/j.psep.2021.05.017.

[ref10] Zhang X.; Gao J.; Jia G.; Zhang J. Study on the influence mechanism of air leakage on gas extraction in extraction boreholes. Energy Explor. Exploit. 2022, 40 (5), 1344–1359. 10.1177/01445987211070664.

[ref11] Zhang Y.; Hu S.; Xia T.; Liu Y.; Pan Z.; Zhou F. A novel failure control technology of cross-measure borehole for gas drainage: A case study. Process Saf. Environ. Prot. 2020, 135, 144–156. 10.1016/j.psep.2019.12.003.

[ref12] Jiang L.; Bao R.; Lei C. Expansion Characteristics and Creep Test of New Curing Expansion Material for Gas Extraction Boreholes. Processes 2024, 12 (2), 293 10.3390/pr12020293.

[ref13] Li B.; Jian W.; Zhang J.; Wang B.; Zhu D.; Wang N. Research Progress and Discussion on Modified Cement-Based Borehole Sealing Materials for Mining. ACS Omega 2023, 8 (15), 13539–13550. 10.1021/acsomega.3c01113. 37091402 PMC10116528

[ref14] Yu S.; Su X.; Song J.; Wang Q.; You Z. The Hole Sealing Technology of Solid–Liquid Materials with Three Pluggings and Two Injections for Gas Extraction Hole in the Coal Mine. ACS Omega 2022, 7 (48), 43847–43855. 10.1021/acsomega.2c05001. 36506120 PMC9730471

[ref15] Yue J.; Ma Y.; Wang Z.; Zhang X.; Wang L.; Shen X. Characteristics of water migration during spontaneous imbibition in anisotropic coal. Energy 2023, 263, 126054 10.1016/j.energy.2022.126054.

[ref16] Zhang J.; Zhai C.; Zhong C.; Xu J.; Sun Y. Investigation of sealing mechanism and field application of upward borehole self-sealing technology using drill cuttings for safe mining. Saf. Sci. 2019, 115, 141–153. 10.1016/j.ssci.2019.02.007.

[ref17] Fu J.; Wang D.; Li X.; Wang Z.; Shang Z.; Jiang Z.; Wang X.; Gao X. Experimental Study on the Cement-Based Materials Used in Coal Mine Gas Extraction for Hole Sealing. ACS Omega 2021, 6 (32), 21094–21103. 10.1021/acsomega.1c02911. 34423217 PMC8375108

[ref18] Hao M.; Song X.; Shi H.; Zhou F.; Shi B. Experimental investigation of cement-based sealing materials for degasification using coal-bed methane drainage system. Mater. Express 2018, 8 (2), 113–122. 10.1166/mex.2018.1424.

[ref19] Hu S.; Hao G.; Feng G.; Guo H.; Wu D. A method for improving the methane extraction concentrations of in-seam boreholes. Fuel 2020, 265, 117006 10.1016/j.fuel.2020.117006.

[ref20] Fu J.; Li X.; Wang Z. A novel sealing material and a bag-grouting sealing method for underground CBM drainage in China. Constr. Build. Mater. 2021, 299, 124016 10.1016/j.conbuildmat.2021.124016.

[ref21] Zhang Y.; Zou Q.; Guo L. Air-leakage Model and sealing technique with sealing–isolation integration for gas-drainage boreholes in coal mines. Process Saf. Environ. Prot. 2020, 140, 258–272. 10.1016/j.psep.2020.03.024.

[ref22] Liu Q.; Cheng Y.; Yuan L.; Fang Y.; Shi D.; Kong S. A new effective method and new materials for high sealing performance of cross-measure CMM drainage boreholes. J. Nat. Gas Sci. Eng. 2014, 21, 805–813. 10.1016/j.jngse.2014.10.023.

[ref23] Xiang X.; Zhai C.; Xu Y.; Yu X.; Xu J. A flexible gel sealing material and a novel active sealing method for coal-bed methane drainage boreholes. J. Nat. Gas Sci. Eng. 2015, 26, 1187–1199. 10.1016/j.jngse.2015.08.016.

[ref24] Wang G.; Fan C.; Xu H.; Liu X.; Wang R. Determination of long horizontal borehole height in roofs and its application to gas drainage. Energies 2018, 11 (10), 2647 10.3390/en11102647.

[ref25] Gao M.; Xie J.; Guo J.; Lu Y.; He Z.; Li C. Fractal evolution and connectivity characteristics of mining-induced crack networks in coal masses at different depths. Geomech. Geophys. Geo-Energy Geo-Resour. 2021, 7, 9 10.1007/s40948-020-00207-4.

[ref26] Zhang H.; Cheng Y.; Liu Q.; Yuan L.; Dong J.; Wang L.; Qi Y.; Wang W. A novel in-seam borehole hydraulic flushing gas extraction technology in the heading face: Enhanced permeability mechanism, gas flow characteristics, and application. J. Nat. Gas Sci. Eng. 2017, 46, 498–514. 10.1016/j.jngse.2017.08.022.

[ref27] Niu Y.; Zhang X.; Wang E.; Li Z.; Cheng Z.; Duan X.; Li H.; Wei Y.; Qian J.; Cai G.; et al. A new method of monitoring the stability of boreholes for methane drainage from coal seams. Measurement 2020, 154, 107521 10.1016/j.measurement.2020.107521.

[ref28] Chu T.; Li P.; Chen Y. Risk assessment of gas control and spontaneous combustion of coal under gas drainage of an upper tunnel. Int. J. Min. Sci. Technol. 2019, 29 (3), 491–498. 10.1016/j.ijmst.2018.05.002.

[ref29] Zhang J.; Ruan G.; Bai Y.; Ning T. Mathematical Model and Numerical Simulation Study of the Mining Area with Multiple Air Leakage Paths. Mathematics 2022, 10 (14), 2484 10.3390/math10142484.

[ref30] Mu Y. B. Spontaneous combustion characteristics and air leakage control technology of half isolated island fully mechanized caving face. Adv. Mater. Res. 2013, 718–720, 1639–1644. 10.4028/www.scientific.net/AMR.718-720.1639.

[ref31] Hu X.; Su M.; Dong H.; Chen X.; Wang F.; Luo C.; Yu S. Study on coal spontaneous combustion dangerous zone under different air leakage in the shallow buried coal seam fissure. Combust. Sci. Technol. 2023, 1–25. 10.1080/00102202.2023.2233052.

[ref32] Huang X.; Liu Q.; Shi K.; Pan Y.; Liu J. Application and prospect of hard rock TBM for deep roadway construction in coal mines. Tunnelling Underground Space Technol. 2018, 73, 105–126. 10.1016/j.tust.2017.12.010.

[ref33] Qin B.; Wei G.; Lou Z.; Wang Z.; He F.; He W.; Zhu H. A new cross-borehole hydraulic caving technique in the coal seam with a soft layer for preventing coal and gas outbursts during coal roadway excavation. Energy Sci. Eng. 2020, 8 (4), 1120–1134. 10.1002/ese3.572.

[ref34] Shao S.; Wu C.; Hao M.; Song X.; Su X.; Wang W.; Li G.; Shi B. A novel coating technology for fast sealing of air leakage in underground coal mines. Int. J. Min. Sci. Technol. 2021, 31 (2), 313–320. 10.1016/j.ijmst.2020.08.004.

[ref35] Ba Q. In Research on Gas Leakage Mechanism and Detection Technology of Coal Mine Gas Drainage, IOP Conference Series: Earth and Environmental Science; IOP Publishing, 2020.

[ref36] Wang G.; Guo H.; Sun L. L. Study on Distribution of Gas Concentration in Air Return Corner Based on Co-Kriging Interpretation. Adv. Mater. Res. 2013, 610, 1188–1193. 10.4028/www.scientific.net/AMR.610-613.1188.

[ref37] Liu H.; Cheng Y. The elimination of coal and gas outburst disasters by long distance lower protective seam mining combined with stress-relief gas extraction in the Huaibei coal mine area. J. Nat. Gas Sci. Eng. 2015, 27, 346–353. 10.1016/j.jngse.2015.08.068.

[ref38] Zhao D.; Liu J.; Pan J.-t. Study on gas seepage from coal seams in the distance between boreholes for gas extraction. J. Loss Prev. Process Ind. 2018, 54, 266–272. 10.1016/j.jlp.2018.04.013.

[ref39] Hu Y.; Wang G.; Chen J.; Liu Z.; Fan C.; Cheng Q. Prediction of gas emission from floor coalbed of steeply inclined and extremely thick coal seams mined using the horizontal sublevel top-coal caving method. Energy Sources, Part A 2020, 1–17. 10.1080/15567036.2020.1733143.

[ref40] Zheng C.; Li H.; Kizil M.; Jiang B.; Xue S.; Yang W.; Chen Z. Performance enhancement of horizontal underground-to-inseam gas drainage boreholes with double-phase-grouting sealing method for coal mining safety and clean gas resource. J. Nat. Gas Sci. Eng. 2020, 76, 103179 10.1016/j.jngse.2020.103179.

